# Anti-cancer stemness and anti-invasive activity of bitter taste receptors, TAS2R8 and TAS2R10, in human neuroblastoma cells

**DOI:** 10.1371/journal.pone.0176851

**Published:** 2017-05-03

**Authors:** Yoona Seo, Yoo-Sun Kim, Kyung Eun Lee, Tai Hyun Park, Yuri Kim

**Affiliations:** 1 Department of Nutritional Science and Food Management, Ewha Womans University, Seoul, Republic of Korea; 2 School of Chemical and Biological Engineering, Seoul National University, Seoul, Republic of Korea; Swedish Neuroscience Institute, UNITED STATES

## Abstract

Neuroblastoma (NB) originates from immature neuronal cells and currently has a poor clinical outcome. NB cells possess cancer stem cells (CSCs) characteristics that facilitate the initiation of a tumor, as well as its metastasis. Human bitter taste receptors, referred to as TAS2Rs, are one of five types of basic taste receptors and they belong to a family of G-protein coupled receptors. The recent finding that taste receptors are expressed in non-gustatory tissues suggest that they mediate additional functions distinct from taste perception. While it is generally admitted that the recognition of bitter tastes may be associated with a self-defense system to prevent the ingestion of poisonous food compounds, this recognition may also serve as a disease-related function in the human body. In particular, the anti-cancer stemness and invasion effects of TAS2Rs on NB cells remain poorly understood. In the present study, endogenous expression of *TAS2R8* and *TAS2R10* in SK-N-BE(2)C and SH-SY5Y cells was examined. In addition, higher levels of *TAS2R8* and *TAS2R10* expression were investigated in more differentiated SY5Y cells. Both TAS2Rs were up-regulated following the induction of neuronal cell differentiation by retinoic acid. In addition, ectopic transfection of the two TAS2Rs induced neurite elongation in the BE(2)C cells, and down-regulated CSCs markers (including DLK1, CD133, Notch1, and Sox2), and suppressed self-renewal characteristics. In particular, TAS2RS inhibited tumorigenicity. Furthermore, when TAS2Rs was over-expressed, cell migration, cell invasion, and matrix metalloproteinases activity were inhibited. Expression levels of hypoxia-inducible factor-1α, a well-known regulator of tumor metastasis, as well as its downstream targets, vascular endothelial growth factor and glucose transporter-1, were also suppressed by TAS2Rs. Taken together, these novel findings suggest that *TAS2Rs* targets CSCs by suppressing cancer stemness characteristics and NB cell invasion, thereby highlighting the chemotherapeutic potential of bitter taste receptors.

## Introduction

Classically, taste perception has been characterized as a flavor-dependent chemosensory system of the taste buds that are located on papillae in the tongue. Moreover, the five basic tastes, including sweet, umami, sour, salty, and bitter, are detected by specialized sensory cells that are localized in the tongue [1]. Among these cells, the mechanisms by which sweet, umami, and bitter tastes have been shown to involve the interactions of small molecules with specific types of G-protein-coupled receptors (GPCRs). GPCRs are a super-family of trans-membrane receptors that respond to diverse extracellular stimuli such as neurotransmitters, light, taste, and smell [2].

Human bitter taste receptors, referred to as TAS2Rs, are a group of ~ 25 chemosensory receptors that respond to bitter substances [3]. Interestingly, recent studies have demonstrated that TAS2Rs are also expressed in non-gustatory tissues, including gastrointestinal, cardiovascular, pulmonary, reproductive, immune, and central nervous system tissues. These findings suggest that TAS2Rs mediate functions that are distinct from a role in detection of taste [4]. Putative functions of TAS2Rs have also been related to various diseases, including severe asthma and cancer [5–7]. In breast and pancreatic cancers, targeting of TAS2Rs has shown potential to serve as a novel anti-cancer strategy [6, 8]. To date, there have been limited studies of bitter taste receptors, although it is generally accepted that sensing of bitter taste are associated with a self-defense system by which humans can protect themselves against the ingestion of potentially harmful, dangerous, and toxic substances [9].

Neuroblastoma (NB) is a type of cancer that develops during the very early stages of an embryo or fetus and originates in immature neuronal cells. Most of cases of NB occur in infancy and in children younger than 10 years old age. In fact, NB is the most prevalent cancer type diagnosed in infants younger than 1 year-old of age [10, 11]. In NB cell lines, three distinct cell types have been identified on the basis of phenotype and gene expression pattern: neuroblastic/neuroendocrine precursor (N-type), substrate-adherent/Schwannian (S-type), and intermediate (I-type) [12, 13]. The latter cells represent the most immature and malignant population of NB cells and they exhibit intermediate properties of both N- and S-type cells [12]. Due to significant similarities in the gene expression profiles of malignant NBs, one of I-type cells, BE(2)C cells have been used to as an *in vitro* model for studies of potential therapeutic targets of NB, particularly as a model of cancer stem cells (CSCs).

It has been demonstrated that sub-populations of cancer cells exhibit representative characteristics of CSCs, including differentiation, self-renewal potential, and tumorigenicity [14]. Correspondingly, CSCs have been shown to be responsible for tumor growth, metastasis, and resistance to chemotherapy and radiotherapy [15]. Therefore, key therapeutic strategies for targeting CSCs could represent an effective treatment for malignant cancer. NBs originate in the peripheral sympathetic nervous system, yet they have the capacity to metastasize to distant organs, including to the adrenal medulla, abdomen, chest, neck, bone, and bone marrow [15]. It is metastasis events, rather than the primary tumor itself, is responsible for the mortality of NB patients [16]. Cell invasion and migration are two fundamental processes that are required for tumor cells metastasis. During these processes, the secretion of matrix metalloproteinases (MMPs) mediates a degradation of the extracellular matrix (ECM), and this allows cancer cells to enter the blood or lymphatic system [17]. Hypoxia is a condition that facilitates metastasis by regulating cell proliferation, ECM production, and cell adhesion [18]. Correspondingly, hypoxic tumor cells exhibit highly tumorigenicity, poor differentiation, and stem cell characteristics [19].

Among the TAS2Rs, TAS2R8, TAS2R10, TAS2R14, and TAS2R46 have been found to be activated by bitter compounds *in vitro* [20–23]. In addition, several well-known agonists of specific bitter taste receptors have been found to exhibit anti-cancer effects. For example, an allyl isothiocyanate, agonist of TAS2R38 has shown desirable benefits as a cancer chemo-preventive drug [24, 25]. Agonists of TAS2R14, including quercetin and naringenin, have exhibited anti-cancer activities in various cancer types, including NB cells [24, 26, 27]. Recently, it has been reported that differential expression of TAS2Rs were observed in non-cancerous breast epithelial versus breast cancer cells, and TAS2Rs were down-regulated in breast cancer cells [6]. However, the anti-cancer stemness and anti-metastatic effects of TAS2Rs remain poorly understood. For this study, we hypothesized that TAS2Rs are expressed in NB cells and they affect cancer stemness, migration, and/or invasion of NB cells *in vitro* and *in vivo*. In particular, the effect of TAS2Rs on cell differentiation, self-renewal capacity, and tumorigenicity were examined.

## Materials and methods

### Cell culture

Two human NB cell lines, SK-N-BE(2)C (BE(2)C) and SH-SY5Y (SY5Y), were purchased from American Type Culture Collection (ATCC, Rockville, MD, USA). The human embryonic kidney cell line, HEK293, was obtained from Dr. Tai Hyun Park, Seoul National University, Korea.

BE(2)C and SY5Y cells were cultured in 1:1 mixture of Minimum Essential Medium (MEM) and Ham’s F-12 (Welgene, Daegu, Korea). HEK293 cells were cultured in a 1:1 mixture of MEM and high glucose Dulbecco’s Modified Eagle’s Medium (DMEM, Welgene). All media were supplemented with 10% fetal bovine serum (FBS; Hyclone, Logan, UT, USA) and 1% penicillin (100 U/mL), and 100 μg/mL streptomycin (Invitrogen, Carlsbad, CA, USA). To induce hypoxia conditions, 1 mM sodium pyruvate (Junsei Chemical, Tokyo, Japan) and 25 mM HEPES (Biosesang, Seongnam, Korea) were added to standard growth medium at pH 7.4. Cell cultures were maintained in an incubator at 37°C with 5% CO_2_ atmosphere.

All-*trans*-retinoic acid (RA) and cobalt(II) chloride hexahydrate (CoCl_2_·6H_2_O) were purchased from Sigma-Aldrich (St. Louis, MO, USA). RA (1 μM) was used to induce neuronal differentiation of NB cells, and CoCl_2_·6H_2_O (100 μM) was used to mimic hypoxic conditions at 21% O_2_. These reagents were diluted with cell culture medium just prior to each assay under the dim light. To activate TAS2R8 and TAS2R10, denatonium benzoate (Sigma-Aldrich) was used in calcium mobilization assays.

### Animal tongue, liver, and brain tissues

Mouse tissue experiments were performed using 5-week-old male C57BL/6 mice (Central Lab Animal Inc, Seoul, Korea). Mouse tongue and liver and brain (cerebellum and hypothalamus) tissues were extracted and mRNA was isolated. Levels of mouse *TAS2R108* (human *TAS2R8*) and mouse *TAS2R103* (human *TAS2R10*) mRNA were assayed in all tissues and GAPDH was used as a loading control.

### Construction and transfection of the TAS2Rs plasmid vectors

Recombinant plasmid constructs, pcDNA3(+TAS2R8) and pcDNA3(+TAS2R10), were constructed and kindly gifted by Dr. Tai Hyun Park, Seoul National University, Korea. Briefly, cDNA PCR products for *TAS2R8* and *TAS2R10* were digested with the restriction enzymes, SmaI and XhoI for TAS2R8, and EcoRI and XhoI for TAS2R10, respectively (Takara Bio, Mountain View, CA, USA). A FLAG-tagged pcDNA3 vector was digested with EcoRV and XhoI for TAS2R8, and EcoRI and XhoI for TAS2R10. The digested PCR products were separated by electrophoresis and extracted and purified from agarose gels for ligation into the pcDNA3 vector. These recombinant plasmids were amplified by transformation with *dh5-α Escherichia coli* competent cells. Following selection of transformed cells on solid LB medium (BD Bioscience, San Jose, CA, USA) supplemented with ampicillin (Amresco, Solon, OH, USA), plasmid DNA was recovered from a subset of colonies for sequencing. BE(2)C cells were then seeded into 6-well plates and transfected with pcDNA3 (Empty vector), pcDNA3+TAS2R8, and pcDNA3+TAS2R10 recombinant plasmids for 48 h until the cells reached to 70–80% confluence. After the transfection, expression and function of these receptors were confirmed using Western blot assays with Flag tag and calcium assay. This transient transfection was performed in all *in vitro* experiments.

### Calcium mobilization assay

A Fura-2 QBT^™^ Calcium Kit (Molecular Devices, Sunnyvale, CA, USA) was used according to the manufacturer’s instructions. Briefly, HEK293 cells (8 x 10^4^) and BE(2)C cells (5 x 10^4^) were seeded into 96-well clear bottom black plates (Corning Inc., Corning, NY, USA). The next day, HEK293 cells and BE(2)C cells were transfected with pcDNA+TAS2R8 and pcDNA+TAS2R10 and were maintained for 48 h. Activation of each receptor in each cell line was detected by measuring the ratio of the fluorescent intensities immediately after the application of a common bitter taste receptor agonist, denatonium benzoate. The signals were measured using a Flex Station III fluorescence plate reader (Molecular Devices) at 510 nm, following excitations of 340 nm for the Ca^2+^-bound Fura-2 dye and at 380 nm for unbound Ca^2+^.

### Quantitative real-time PCR analysis

Total RNA was isolated using TRizol reagent (Invitrogen) and the first-strand cDNAs were synthesized by reverse transcription using a RevertAid reverse transcriptase (Thermo Scientific, Waltham, MA, USA). The cDNA samples were combined with 2X SYBR Green PCR Master Mix (Qiagen) and the Quantitative real-time PCR was performed using Rotor-Gene^®^ Q (Qiagen, Hilden, Germany) according to the following conditions: initiation at 95°C (5 min), denaturation at 95°C (15 sec), and annealing and extension at 60°C (10 sec). The sequences of the primers we used are: (1) human DLK1: (forward) 5′-CTG AAG GTG TCC ATG AAA GAG-3′ and (reverse) 5′-GCT GAA GGT GGT CAT GTC GAT-3′; (2) human CD133: (forward) 5′-TGG ATG CAG AAC GGT ACA AC-3′ and (reverse) 5′-ATA CCT GCT ACG ACA GTC GT-3′; (3) human Notch1: (forward) 5′-GAG GCG TGG CAG ACT ATG C-3′ and (reverse) 5′-CTT GTA CTC CGT CAG CGT GA-3′; (4) human Sox2: (forward) 5′-CAA GAT GCA CAA CTC GGA GA-3′ and (reverse) 5′-GCT TAG CCT CGT CGA TGA AC-3′; (5) human TAS2R8: (forward) 5′-GAA GAC ATT AAG GCA GGT GGT-3′ and (reverse) 5’-CGC CAG AAT TTG TTT GAT CAG TG-3′; (6) human TAS2R10: (forward) 5′-TGA AAT AGC TAA GCC GGT GAG-3′ and (reverse) 5′-ACG TGT AGT GGA AGG CAT CT-3′; (7) mouse TAS2R108 (TAS2R8): (forward) 5′-ATT TGT GTT TGC TGC CTC GG-3′ and (reverse) 5′-GTG ATG GCC AAG CTG AAC AG-3′; (8) mouse TAS2R103 (TAS2R10): (forward) 5′-TCC AAG AAT CAGTAC ACA GGA GT-3′ and (reverse) 5′-AAA GGC TTG CAA ACT GTG GT-3′; (9) human MMP-2: (forward) 5′-CTT CCA AGT CTG GAG CGA TGT-3′ and (reverse) 5′-TAC CGT CAA AGG GGT ATC CAT-3′; (10) human P-selectin: (forward) 5′-GGG GCT CAA CTC ATC TGG TT-3′ and (reverse) 5′-CCT ACA GAA CAC CCG TGA GT-3′; (11) human VEGF: (forward) 5′-GCA CCC ATG GCA GAA GG-3′ and (reverse) 5′-CTC GAT TGG ATG GCA GTA GCT-3′; (12) human GLUT1: (forward) 5′-GAT TGG CTC CTT CTC TGT GG-3′ and (reverse) 5′-TCA AAG GAC TTG CCC AGT TT-3′; (13) human GAPDH: (forward) 5′-AGA AGG CTG GGG CTC ATT TG-3′ and (reverse) 5′-AGG GGC CAT CCA CAG TGT TC-3′; and (14) mouse GAPDH: (forward) 5′-GCC TTC CGT GTT CCT ACC C-3′ and (reverse) 5′-TGC CTG CTT CAC CAC CTT C-3′. As an internal control, *GAPDH* was measured to normalize all of the qRT-PCR data.

### Western blot assay

Western blot assays were performed as previously described [28]. Briefly, cell protein extracts were collected with a RIPA lysis buffer and equal amounts of each extract sample were separated in 8% or 10% sodium dodecyl sulfate (SDS) polyacrylamide gels. After the samples were transferred onto polyvinylidene fluoride (PVDF) membranes (Millipore, Billerica, MA, USA), they were blocked with 5% bovine serum albumin (BSA) or skim milk in Tris-buffered saline/Tween 20 (TBS-T). The blocked membranes were incubated overnight at 4°C with primary antibodies recognizing a Flag(DYKDDDK) tag, phosphorylated (phospho)-ERK, total ERK (Cell Signaling, Danvers, MA, USA), β-tubulin III (Sigma-Aldrich), HIF-1α (Novus Biologicals, Littleton, CO, USA), and α-tubulin (Sigma-Aldrich). The membranes were washed several times and then anti-mouse or anti-rabbit secondary antibodies (Santa Cruz Biotechnology, Santa Cruz, CA, USA) were added as appropriate. After 1 h, an ECL detection system was used to visualize bound antibodies.

### Clonogenic assay

Clonogenic assays were performed as previously described [29]. Briefly, BE(2)C cells (800 cells/well) were plated in 6-well plates. The next day, the cells were transfected with each plasmid including pcDNA (empty vector), pcDNA+TAS2R8, and pcDNA+TAS2R10 as directed by the manufacturer’s instructions. After 48 h, the transfected cells were maintained for an additional 7–10 days. The colonies were stained with crystal violet (Sigma-Aldrich). Plating efficiency (PE, %) was calculated as: the number of stained colonies / the number of seeded cells x 100%.

### Sphere formation assay

To promote sphere formation, BE(2)C cells (3 x 10^4^/well) were seeded into cell culture plates precoated with 1.2% poly-HEMA (Sigma-Aldrich) and maintained with a 1:1 mixture medium of DMEM and F-12 (Welgene) supplemented with 20 ng/mL epidermal growth factor (EGF; PepTech, Rocky Hill, NJ, USA), 40 ng/mL fibroblast growth factor (FGF; PeproTech), 2% B-27 (Invitrogen), 10% BSA (Rdtech Technology) and 2% penicillin-streptomycin (Invitrogen) [29]. The next day, the cells were transfected with each plasmid. After 48 h. the cells were incubated for an additional 10–14 days and the number of spheres containing more than 50 cells was counted and photographed (Nikon Instruments Co. Ltd, Tokyo, Japan).

### Wound-healing assay

To investigate cell migration, wound-healing assays were performed as previously described [30]. Briefly, BE(2)C cells were grown in 6-well plates in a monolayer up to 80–90% confluence. Then 4–6 lines (each approximately 1 mm wide) were scratched in each monolayer with a sterile 200 μL pipette tip. A representative line with similar width was selected in each group and monitored. Dislodged cells were removed by washing with fresh medium, and the remaining cells were transfected with pcDNA3, pcDNA3+TAS2R8, and pcDNA3+TAS2R10. Cell migration was photographed at 24 h and 48 h later to measure the distance migrated by the cells.

### Transwell invasion assay

Cell invasion was examined in transwell invasion assays using 24-well matrigel invasion chambers (Becton Dickinson, Bedford, MA, USA) as described previously [30]. Briefly, 3 x 10^5^ BE(2)C cells were seeded into the rehydrated upper wells with serum-free medium, while the lower chambers were filled with growth medium containing 5% FBS. The cells seeded in the upper wells were transfected with each plasmid. After 48 h, the cells that did not pass through the membrane pores of the upper wells were carefully removed with a cotton swab, while the cells located in the lower wells were fixed with methanol and stained with crystal violet (Sigma-Aldrich). The number of invaded cells in five randomly selected fields for each well was counted.

### Gelatin zymography assay

As previously described [30], gelatin zymography assays were conducted to analyze the enzymatic activity of MMP-2. Briefly, BE(2)C cells were grown in serum-free medium and transfected with each plasmid. After 48 h, each medium was collected and separated in 10% SDS-PAGE gels containing 0.1 mg/mL gelatin. Electrophoresis was performed at 4°C. The gels were then washed with 2.5% Triton X-100 denaturing buffer to remove SDS, and incubated overnight at 37°C in a reaction buffer containing 50 mM Tris-Cl (pH 7.5), 10 mM CaCl_2_, 15 mM NaCl, and 0.2 μM ZnCl_2_. The gels were stained with 0.5% Coomassie Brilliant Blue R-250 (Amresco, Solon, OH, USA) for 1 h on a shaker at room temperature, then were rinsed with a destaining solution [MeOH: H_2_O: acetic acid (40:50:10)] until clear bands of MMP-2 were visible.

### *In vivo* tumorigenicity assay

To establish the stable clones, BE(2)C cells were transfected with pcDNA3, pcDNA3+TAS2R8, and pcDNA3+TAS2R10 plasmids. After 48 h, the medium was replaced with growth medium containing G418 (Invitrogen). After 2 weeks, stable cell lines were established. One cell line was selected to be subcutaneously injected into the flanks of immune deficient, 5-week-old male BALB/c nude mice (Central Lab Animal Inc, Seoul, Korea) (4 x 10^5^ cells/injection). After the tumors were established, tumor size was monitored twice a week using digital calipers. Tumor volume was calculated as: length (mm) x width^2^ (mm^2^) x 0.5. Animal care and experimental protocols for this study were approved by the Animal Care and Use Committee of Ewha Womans University (IACUC approval No: 16–046).

### Statistical analysis

All experimental results are presented as the mean ± standard error of the mean (SEM) from at least three independent experiments. GraphPad PRISM (GraphPad Software, SanDiego, CA, USA) was used to perform statistical significance analyses. One-way analysis of variance (ANOVA) was applied to compare more than three groups. An unpaired two-tailed Student’s *t*-test was used to compare two group data. To compare the tumor incidence, Pearson’s Chi-square test was performed. A *P*-value less than 0.05 was considered statistically significant. All experiment results are from at least three independent experiments.

## Results

### Expression of TAS2Rs and their functionality in denatonium benzoate-induced calcium mobilization assays

**Fig 1:**
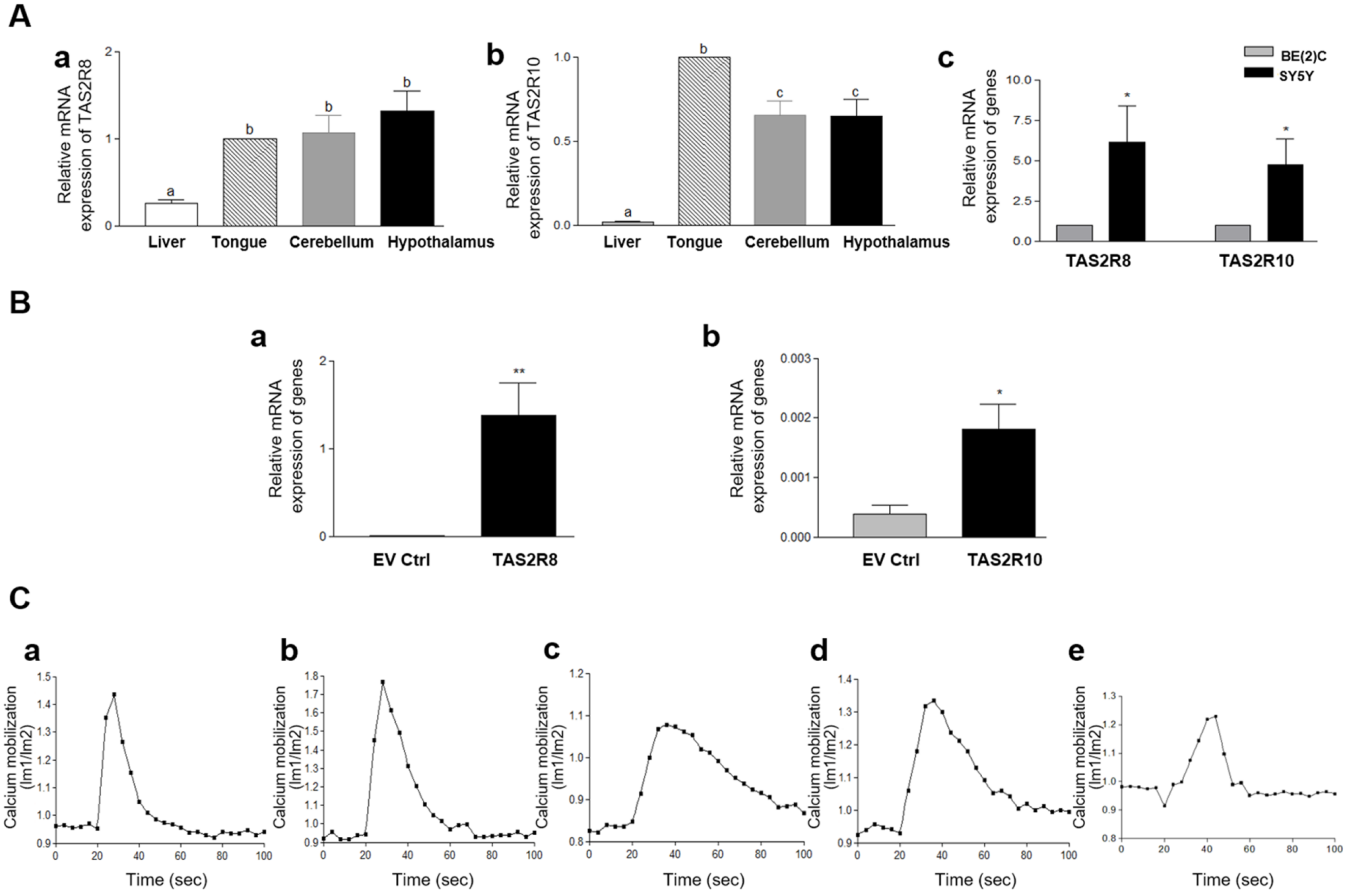
Bitter taste receptors were endogenously expressed and functional in neuroblastoma cell lines. (A) The mRNA levels of *TAS2R8* (a) and *TAS2R10* (b) were detected in mouse liver, tongue, and brain tissues and in BE(2)C and SY5Y cells (c). The bars represent the mean ± SEM (* p < 0.05). One-way analysis of variance (ANOVA) was applied to compare expressions in mouse tissues, and an unpaired two-tailed *t*-test was used to compare two cell lines. (B) mRNA expression of TAS2R8 (a) and TAS2R10 (b) over-expressed BE(2C) cells were analyzed. (C) Denatonium benzoate (0.01 μM) induced an increase in intracellular calcium [Ca^2+^] in HEK293 cells (a-b) and BE(2C) cells (c-e). HEK293 cells were transfected with TAS2R8 (a) and TAS2R10 (b). BE(2)C cells were transfected with an empty vector (EV ctrl) (c), or vectors expressing TAS2R8 (d), or TAS2R10 (e).

Tongue, liver, and brain tissues were collected from the mice. The expression levels detected for *TAS2R8* and *TAS2R10* were much higher in the tongue tissues than in the liver tissues, while the brain tissues examined exhibited different expression patterns for the two receptors (Fig 1Aa & 1Ab). It was determined that the expression of *TAS2R8* was similar in the tongue and brain tissues, yet the expression level of *TAS2R10* was higher in the tongue tissues than in the brain tissues. *TAS2R8* and *TAS2R10* were also highly expressed in the two NB cell lines, BE(2)C and SY5Y. Both *TAS2R8* and *TAS2R10* were expressed at significantly lower levels in the more malignant and less differentiated I-type NB cell line, BE(2)C, compared to the more differentiated N-type cell line, SY5Y cells (Fig 1Ac). These results suggest that TAS2Rs expression was distinct in different tissues, and expression levels of TAS2Rs depend on the differentiation state of cells.

Transient transfection of TAS2R8 and TAS2R10 showed significantly higher mRNA expressions compared to empty vector control (Fig 1Ba and 1Bb). Next, calcium mobilization assays were conducted to evaluate whether ectopic expression of TAS2R8, TAS2R10 in HEK293 cells and BE(2)C cells, and the endogenous expression of these TAS2Rs in BE(2)C cell are functional. In these assays denatonium benzoate (0.01 μM), a common agonist of TAS2R8 and TAS2R10, was applied [24]. Calcium mobilization was detected in each TAS2Rs transfected HEK293 cells and BE(2) cells (Fig 1Ca–1Ce). Although degrees of calcium mobilization were various, the mobilization patterns were similar at time-points at which TAS2R activation occurred and calcium released into the cells. Based on these results, it appears that both ectopically expressed *TAS2R8* and *TAS2R10*, and endogenously expressed both *TAS2R8* and *TAS2R10* were functional.

### Expression of TAS2Rs during neuronal differentiation

**Fig 2:**
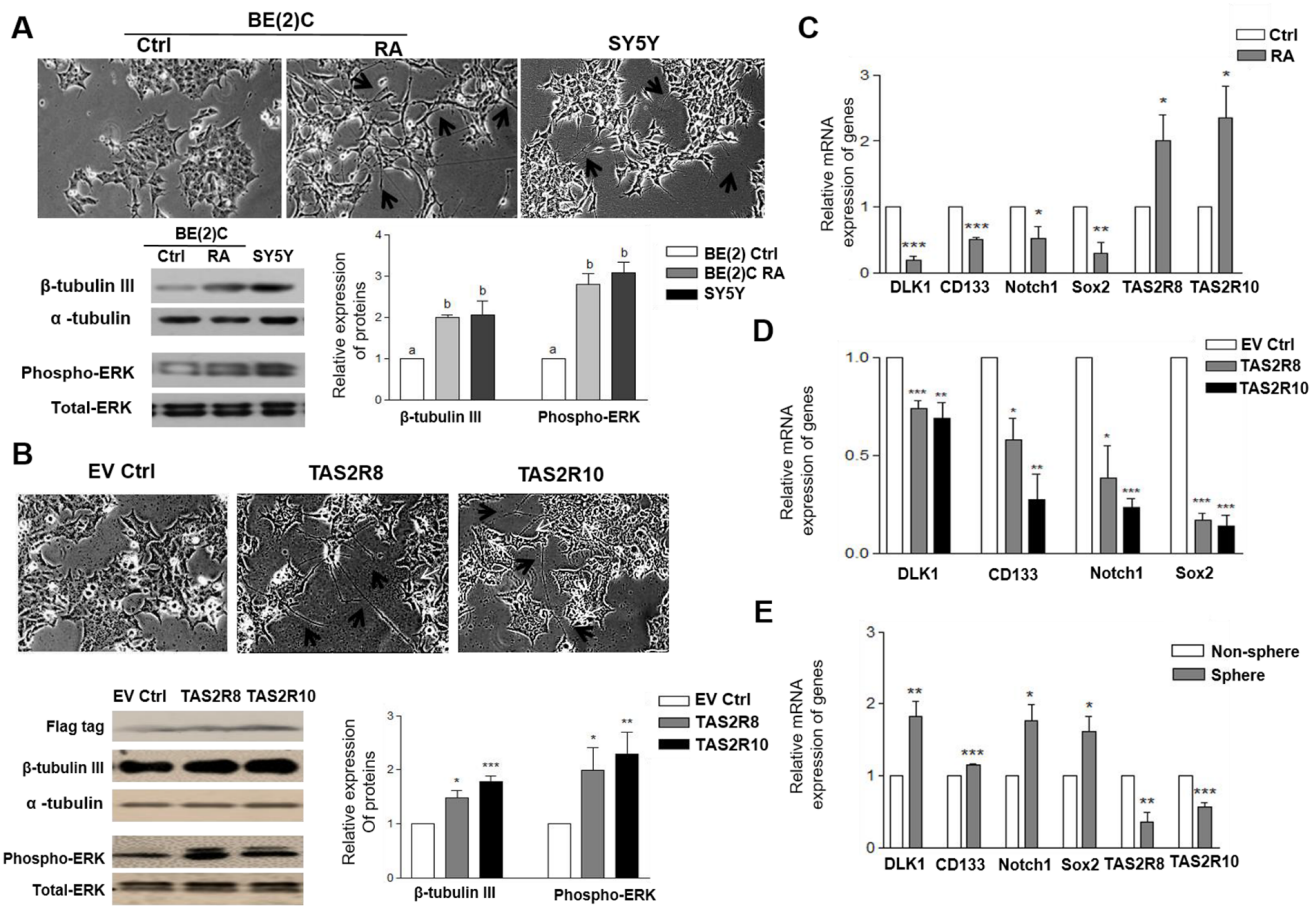
*TAS2R8* and *TAS2R10* were affected during neuronal differentiation, and down-regulated cancer stem cells (CSCs) markers in BE(2)C cells. (A) BE(2)C cells were differentiated over 5 d with RA (1 μM). (A) The morphological features of BE(2)C cells were examined and compared with SY5Y cells. Expression levels of β-tubulin III and phosphorylation of ERK1/2 were also analyzed. (B) BE(2)C cells were transfected with empty vectors (EV ctrl), and vectors expressing *TAS2R8* and *TAS2R10*. Neurite elongation and protein levels of phospho-ERK1/2 and β-tubulin III that were analyzed. Lengthened neurites are indicated with arrows. Flag tag was used to show transfection efficiency. (C) BE(2)C cells were differentiated over 5 d with RA (1 μM). The mRNA levels of various CSC markers and *TAS2R8* and *TAS2R10* were compared with non-treated control cells. (D) BE(2)C cells were transfected with empty vectors, and vectors expressing *TAS2R8* and *TAS2R10*. The mRNA levels of the CSC markers detected were compared with empty vector-transfected control cells. (E) The mRNA levels of CSC markers and *TAS2R8* and *TAS2R10* were compared between non-spheres and formed spheres of BE(2)C cells. An unpaired two-tailed Student’s *t*-test was performed to compare each TAS2R data with the control cells. A *P*-value less than 0.05 was considered statistically significant. (* p < 0.05, ** p < 0.01, *** p < 0.001). Ctrl, control; RA, all-*trans*-retinoic acid; CSCs, cancer stem cells.

To examine whether the expression of TAS2Rs is affected by neuronal cell differentiation, BE(2)C cells were treated with RA (1 μM) for 5 d. Neurite elongation was observed, while up-regulated neuronal differentiation-related markers, including β-tubulin III and phospho-ERK, were also detected (Fig 2A). Similarly, following the over-expression of *TAS2R8* and *TAS2R10* in BE(2)C cells, neurite elongation was observed and levels of β-tubulin III and phospho-ERK1/2 were significantly increased (Fig 2B). Well-differentiated N-type SY5Y cells also showed similar neurite growth and expressions of β-tubulin III and phospho-ERK compared to RA-induced BE(2)C cells.

Next, the effect of TAS2R8 and TAS2R10 on the expression of representative stem cell markers was investigated. After RA treatment, expression of *DLK1*, *CD133*, *Notch1*, and *Sox2* were significantly down-regulated, whereas the levels of *TAS2R8* and *TAS2R10* expression were up-regulated (Fig 2C). Several representative stem cell markers were also significantly down-regulated when the two TAS2Rs were over-expressed in the BE(2)C cells (Fig 2D). Furthermore, gene expression of *TAS2R8* and *TAS2R10* were found to be significantly down-regulated in the spheres that formed in the sphere formation assays (Fig 2E). Taken together, these results suggest that over-expression of *TAS2R8* and *TAS2R10* induces neuronal differentiation and suppresses the expression of CSC markers in BE(2)C cells.

### Effect of TAS2Rs on self-renewal capacity

**Fig 3:**
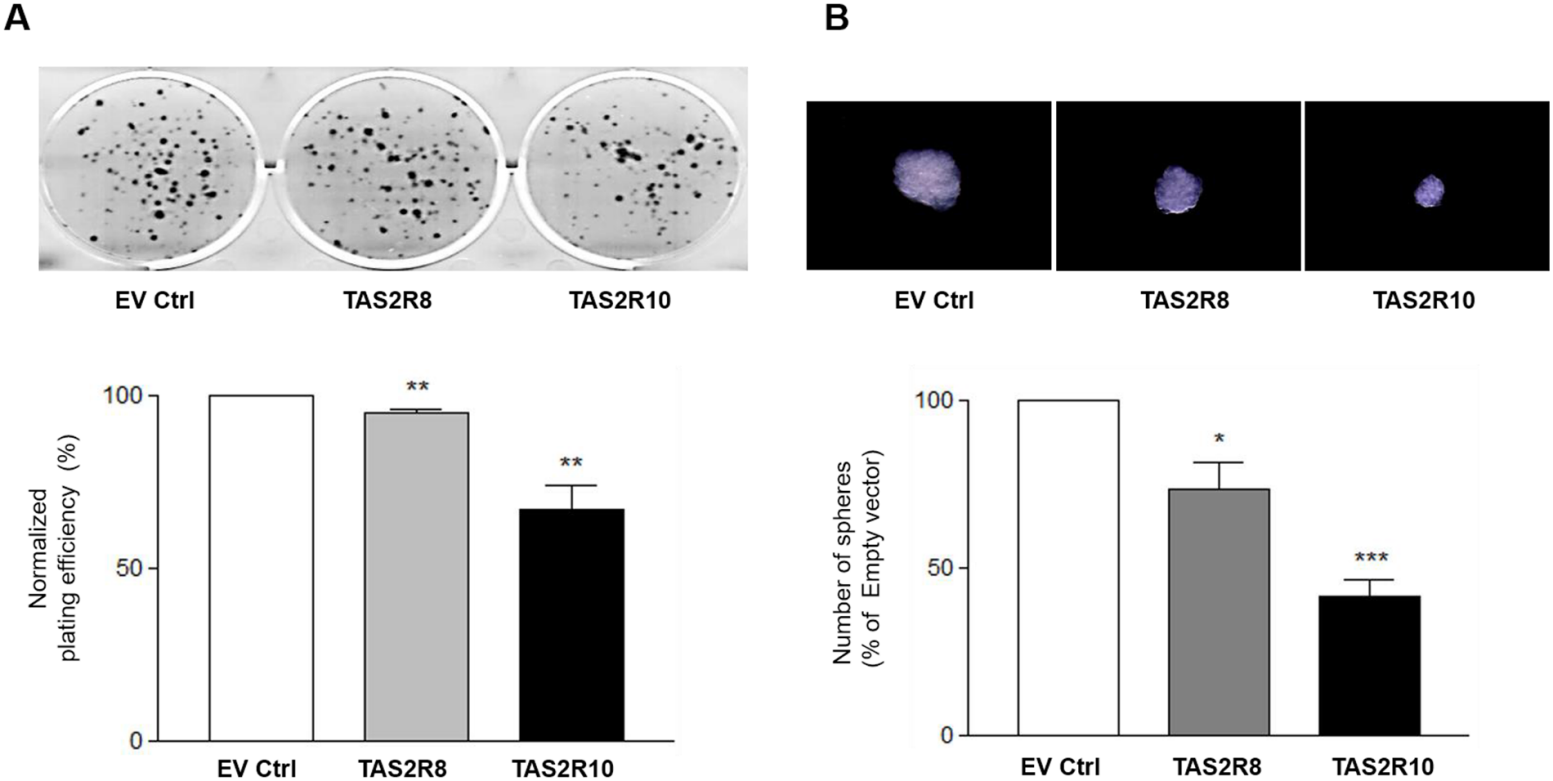
The self-renewal capacity of BE(2)C cells was suppressed following the over-expression of TAS2Rs. BE(2)C cells were transfected with an empty vector (EV ctrl), or vectors expressing *TAS2R8*, or *TAS2R10*. (A) After 48 h, the transfected cells were plated in 6-well plates and cultured for additional 7–10 days. Colonies were then stained and the number of colonies with ≧ 50 cells was counted. (B) The number of spheres was counted. Magnification, x100. The percentages for colony and sphere formation were normalized to that of the empty vector group. An unpaired two-tailed Student’s *t*-test was performed to compare each TAS2R data with the control cells. A *P*-value less than 0.05 was considered statistically significant (* p < 0.05, ** p < 0.01, *** p < 0.001).

Self-renewal capacity is one of characteristics of CSCs, and it represents a physiological effect of cancer stemness [29]. Both clonogenic assays and sphere formation assays were performed to measure self-renewal potential following the ectopic over-expression of *TAS2R8* and *TAS2R10*. Both clonogenic formation (Fig 3A, *p* < 0.0061 and *p* < 0.003, respectively) and sphere formation (Fig 3B, *p* < 0.0268 and *p* < 0.0062, respectively) were significantly suppressed. Taken together, these data indicate that TAS2Rs play an important role in inhibiting the self-renewal potential of NB cells, and also in eliminating NB stem-like cells.

### Effect of TAS2Rs on tumorigenicity in a xenograft model

At the time of sacrifice, 10 out of 10 empty vector control (EV Ctrl) mice had developed tumors, whereas 2 out of 10 TAS2R8 and 7 out of 10 TAS2R10 group had developed tumors (Table 1). This result suggests that over-expression of TAS2R8 and TAS2R10 suppresses tumor incidence by 80% and 30% compared to the control EV group. Tumor volumes and weights were tended to be decreased by over-expression of each TAS2Rs, however they were not statistically significant.

**Table 1:** Effect of over-expressed *TAS2R8* and *TAS2R10* on tumor incidence, final tumor volume, and tumor weight.

Group	Tumor incidence*	Final Tumor volume (mm^3^)	Final Tumor weight (mg)
EV Ctrl	10 / 10	598.1 ± 330.2	278 ± 138.7
TAS2R8	2 / 10	7.05 ± 5.1	4.0 ± 0.3
TAS2R10	7 / 10	173.8 ± 69.7	107.0 ± 42.1

* Tumor incidence comparison among groups was conducted by chi-square test (*P<0*.*05)*. Final tumor volume and weight were presented as the mean ± SEM. An unpaired two-tailed Student’s *t*-test was performed by comparing with control. EV Ctrl; empty vector control

**Fig 4:**
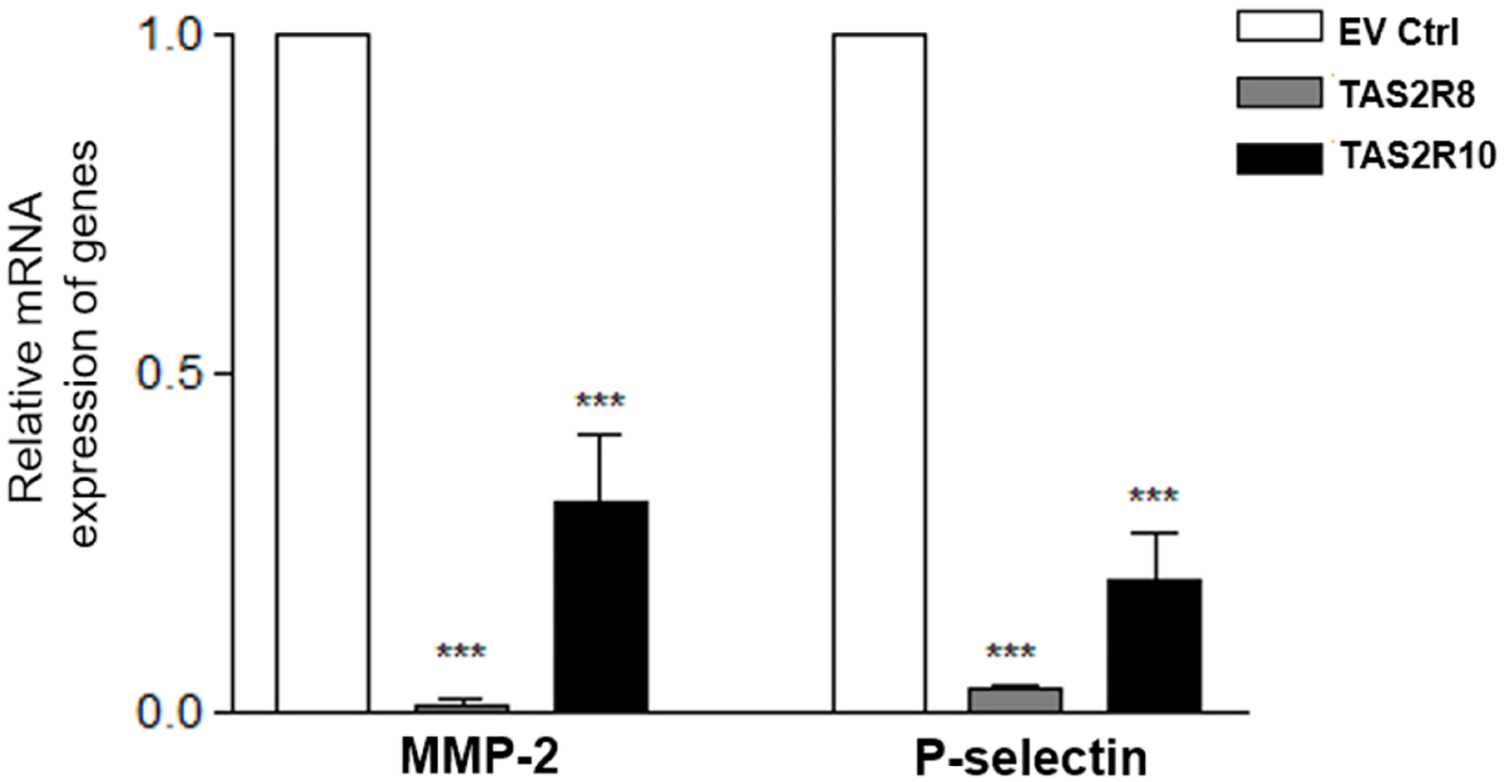
Over-expression of *TAS2R8* and *TAS2R10* suppressed *MMP-2* and *P-selectin* expression. The markers of adhesion, mRNA expressions of *MMP-2* and *P-selectin* were analyzed. An unpaired two-tailed Student’s *t*-test was performed to compare each TAS2Rs tumor group with the empty vector control (EV Ctrl) group. A *P*-value less than 0.05 was considered statistically significant. (* p < 0.05, ** p < 0.01, *** p < 0.001).

It has been reported that adhesive potential plays an important role in the dissemination of metastatic tumors [31]. To examine whether TAS2Rs affect adhesion molecules in the tumor tissues, expression of MMP-2 and P-selectin were analyzed. Both *MMP-2* and *P-selectin* mRNA levels were down-regulated in both of the TAS2Rs over-expressed groups compared to the EV Ctrl group (Fig 4). MMP-2 destructs mesenchymal collagen or the ECM together with adhesion molecules in the process of tumor invasion and metastasis [32]. Thus, these results suggest that TAS2R over-expression can suppress breakdown of the ECM by inhibiting MMP-2, and it can also inhibit P-selectin to affect the adhesive potential of cancer metastasis, which is consistent with the *in vitro* results obtained.

### Inhibition of NB cell migration and invasion

**Fig 5:**
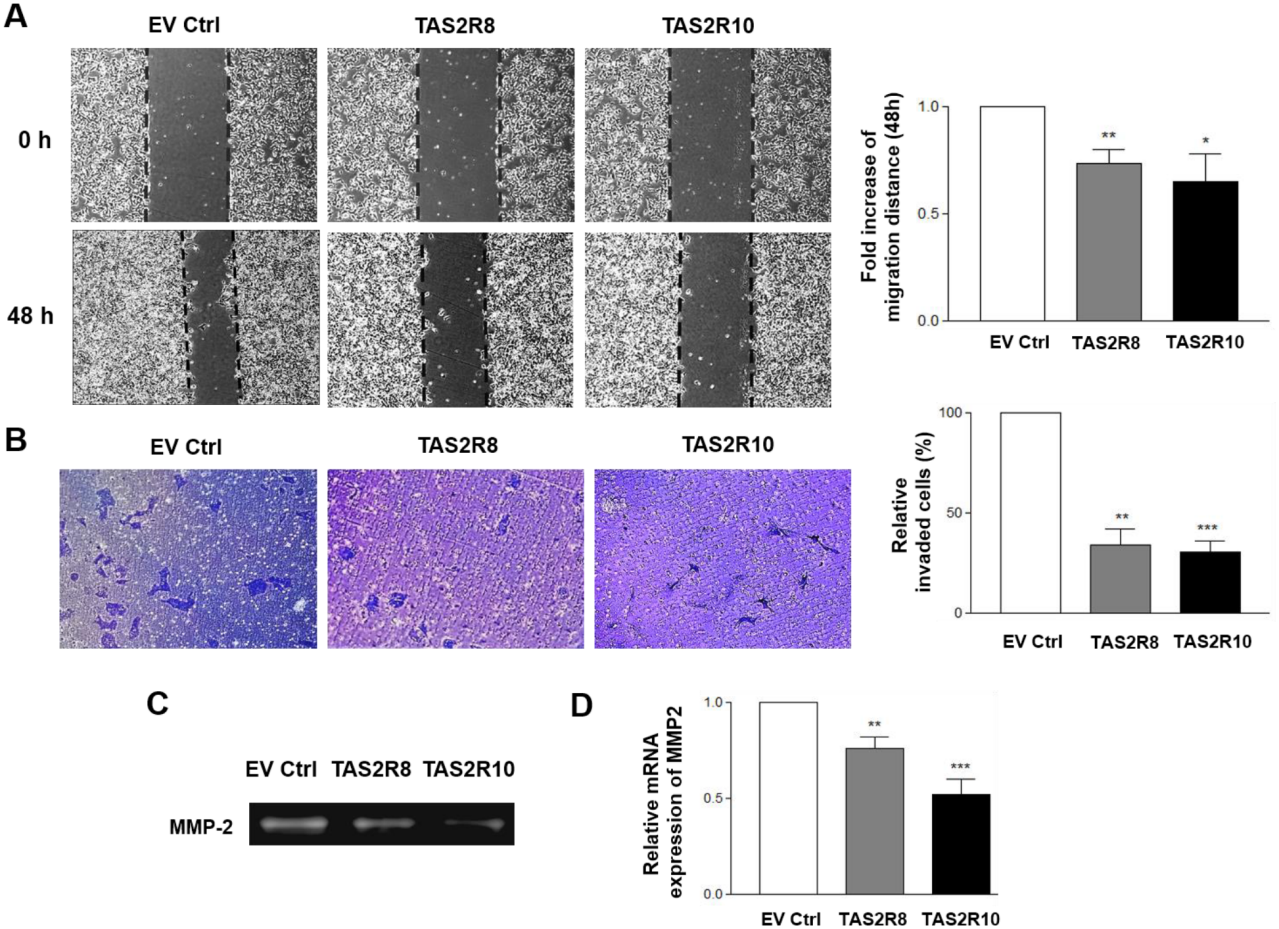
Migration and invasion were suppressed following the over-expression of *TAS2R8* and *TAS2R10* in the BE(2)C cells. BE(2)C cells were transfected with empty vector (EV ctrl), *TAS2R8*, and *TAS2R10*. (A) Cell migration was analyzed in wound-healing assays. Representative images of the migration assay results are shown (left panel). The fold increase in migration distance was compared with the empty vector group (right panel). (B) Cell invasion was evaluated in Transwell assays. Representative images of the invasion assays are shown (left panel). The number of invading cells was counted and the percentage of invading cells in each group was normalized to that of the empty vector group (right panel). (C-D) After *TAS2R8* and *TAS2R10* were over-expressed, MMP enzymatic activity and gene expressions were analyzed. (C) Enzyme activity of MMP-2 was detected in zymography assays. (D) The mRNA level of *MMP-2* was compared with the control group. An unpaired two-tailed Student‘s *t*-test was performed to compare each TAS2R data with the control cells. A *P*-value less than 0.05 was considered statistically significant (* p < 0.05, ** p < 0.01, *** p < 0.001).

It has been reported that the metastatic potential of tumor cells increases under hypoxic conditions, and this is a driving force for the metastasis of cancer cells to other organs [33]. To examine whether TAS2Rs affect metastatic potential, migration and invasion assays were conducted with BE(2) cells over-expressing *TAS2R8* and *TAS2R10*. In the wound-healing assays performed, both sets of transfected BE(2)C cells migrated approximately 30% more slowly compared to the cells that were transfected with an empty vector (EV) (TAS2R8, *p* < 0.0065; TAS2R10, *p* < 0.0386) (Fig 5A). In the transwell invasion assays that were conducted, the invasive potential of the two TAS2Rs was significantly decreased compared to the EV group (Fig 5B; TAS2R8, *p* < 0.0014; TAS2R10, *p* < 0.0002). Taken together, these results indicate that over-expression of TAS2R8 or TAS2R10 suppresses the metastatic potential of BE(2)C cells by inhibiting their ability to migrate and invade.

MMPs degrade macromolecules of the ECM to promote the migration and invasion of cancer cells [34, 35]. Following the over-expression of *TAS2R8* and *TAS2R10*, gelatin zymography and qRT-PCR assays were performed to detect whether over-expression of these TAS2Rs affect the activity and expression of MMP2. It was observed that the activity of MMP-2 (Fig 5C), as well as the expression of *MMP-2* (Fig 5D), were decreased following TAS2R over-expression. Therefore, it appears that over-expression of TAS2Rs inhibits the metastasis potential by suppressing the activity and expression of MMPs.

### Regulation of HIF-1α by TAS2Rs under hypoxic conditions

**Fig 6:**
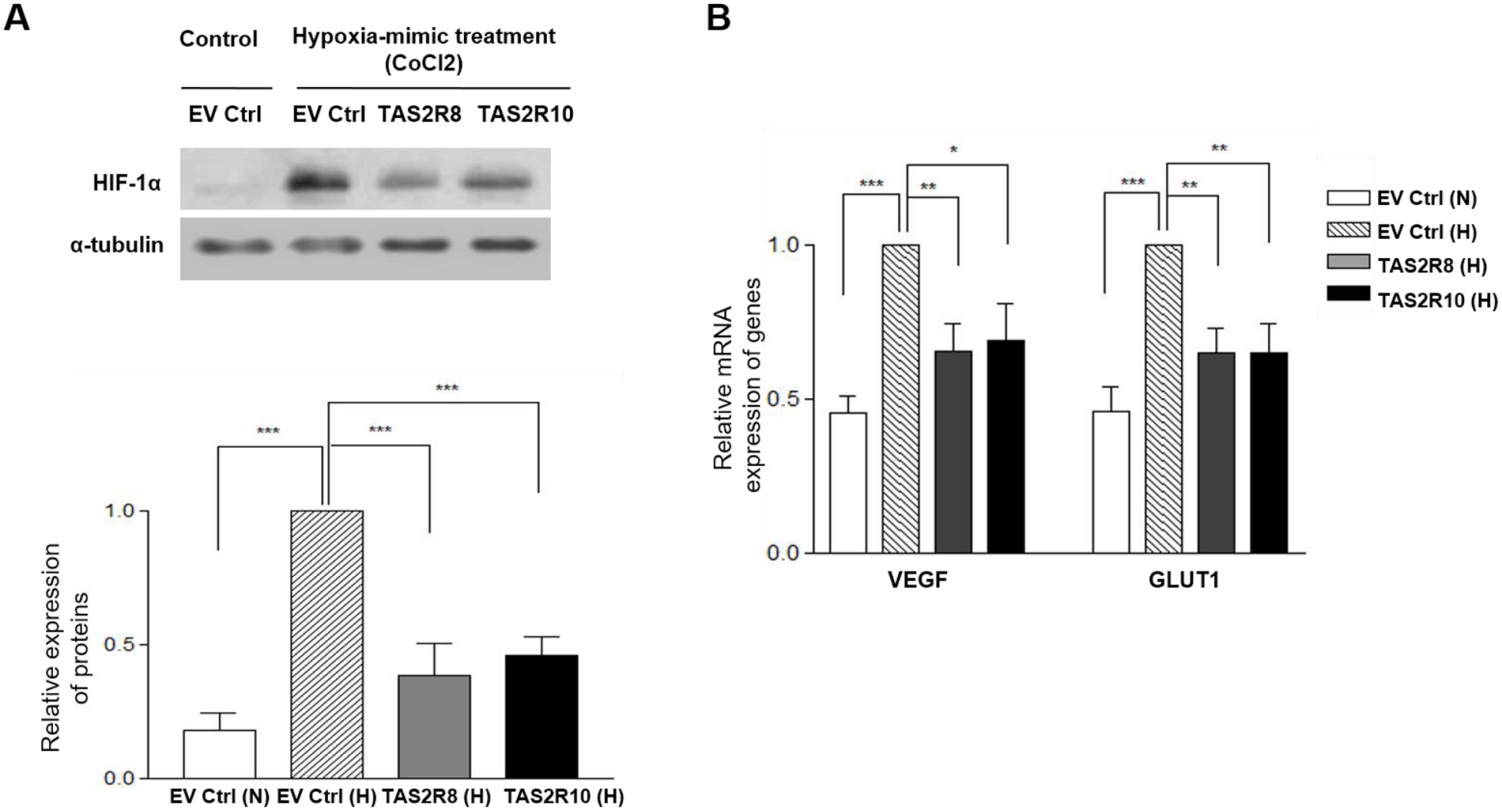
Over-expression of *TAS2R8* and *TAS2R10* suppressed HIF-1α-mediated regulation of *VEGF* and *GLUT1*. (A) After each TAS2Rs construct was over-expressed, each set of BE(2)C cells was treated with CoCl_2·_6H_2_O. After 24 h, protein levels of HIF-1α were compared with the levels detected in the cells that were transfected with an empty vector (EV) and maintained under hypoxic conditions. (B) The mRNA levels of two genes downstream of HIF-1α, *VEGF* and *GLUT1*, were compared with the control group. An unpaired two-tailed Student’s *t*-test was performed to compare each TAS2R data with the control cells. A *P*-value less than 0.05 was considered statistically significant. (* p < 0.05, ** p < 0.01, *** p < 0.001). N, normoxia; H, hypoxia.

HIF-1α is a well-known transcription factor that controls and mediates the metastasis process of tumors by regulating downstream genes such as *VEGF* and *GLUT1* [28]. To investigate whether TAS2Rs regulate the expression of HIF-1α and its related genes, Western blot and qRT-PCR assays were performed. HIF-1α expression was strongly detected following CoCl_2_-induced hypoxia, yet was not detected under normoxic conditions. In addition, over-expression of. *TAS2R8* and *TAS2R10* significantly down-regulated HIF-1α expression compared to the EV Ctrl under CoCl_2_-induced hypoxic conditions (Fig 6A). Furthermore, expressions levels of HIF-1α-downstream genes were affected, with *VEGF* and *GLUT1* up-regulated under CoCl_2_-induced hypoxic conditions, and the same genes were down-regulated following over-expression of *TAS2R8* and *TAS2R10* (Fig 6B). These results suggest that TAS2Rs contribute to the regulation of hypoxia-related gene expression.

## Discussion

The results of the present study demonstrate that *TAS2R8* and *TAS2R10* are endogenously expressed in NB cancer cells and they play noticeable roles in inhibiting the stemness, migration, and invasion of these cancer cells. The effects of these two TAS2Rs on cancer stemness were identified by examining CSC marker expression, self-renewal potentials, and tumorigenicity. In addition, the anti-metastatic effect of these TAS2Rs was evaluated in cell migration, invasion potential, and MMP and HIF1-α expression assays of BE(2)C cells over-expressing *TAS2R8* and *TAS2R10*.

In humans, bitter tastes are detected by 25 GPCRs that constitute the hTAS2R gene family [36]. GPCRs have been utilized as therapeutic targets in various diseases, and these drugs currently account for ~30% of the approved medicines available for disease treatment. However, these drugs have been limited to non-chemosensory receptors [37]. Based on recent reports that taste receptors are expressed in non-gustatory tissues and organs, it has been hypothesized that TAS2Rs mediate additional functions [4]. Correspondingly, it has been demonstrated that GPCRs are involved in neurotransmitter function and in the regulation of neuronal and hormone signaling. Furthermore, impairment of this signaling by these TAS2Rs potentially contributes to CNS-related disorders, including Alzheimer’s disease, schizophrenia, and Parkinson’s [38, 39]. In rats, taste-transducing molecules are predominantly located in neurons, and TAS2R expression has been detected in multiple regions of the rat brains, including the brain stem, cerebellum, cortex, and nucleus accumbens [9, 40]. To date, natural ligands for TAS2Rs in the brain have not been reported, although bitter tasting di- and tri-peptides from food have been shown to access the brain via a peptide transporter [41]. In the present study, both *TAS2R8* and *TAS2R10* were found to be endogenously expressed in BE(2)C cells and SY5Y cells, and the level of expression of these TAS2Rs were lower in the less differentiated and more malignant BE(2)C cells compared to the more differentiated SY5Y cells.

When a bitter agonist of TAS2Rs was applied to individuals with asthma, suppressed abnormal contraction of airway smooth muscle, and inhibited release of inflammatory cytokines via mast cell inactivation were observed [5, 42]. Recently, several studies have also described the expression and roles of TAS2Rs in several cancer cell lines. For example, Singh and colleagues discovered the expression of TAS2R4 differed between metastatic breast cancer and non-cancerous epithelial cell lines [6], and Gaida and colleagues demonstrated the expression and localization of TAS2R38 in a pancreatic cancer cell line [8]. However, little is known about the role of TAS2Rs in the cancer stemness, migration, and invasion.

Various bitter phytochemicals have previously been identified as promising cancer therapeutics. However, the mechanistic details associated with specific TAS2Rs have not been fully elucidated. Moreover, although certain well-known TAS2Rs agonists, such as quercetin, have been investigated in the context of anti-cancer related activities [26], it has not been confirmed whether these effects are due to high levels of TAS2Rs expression or the activation of TAS2Rs. In the present study, over-expression of *TAS2R8* and *TAS2R10* induced neuronal cell differentiation, and this was accompanied by ERK phosphorylation and the suppression of various CSC cell markers. These results are consistent with the observation that a bitter substance from willow bark, salicin, which is a TAS2R16 agonist was able to modulate neurite elongation in SH-SY5Y cells [43] and Kong and colleagues also identified an anti-cancer activity of salicin [44].

It is generally accepted that most tumors start from a single population of CSCs which possess a pluripotent capacity [45]. Pluripotency is a critical function of stem cells and it allows these cells to differentiate into various cell types. Consequently, cell differentiation represents a promising target in cancer therapy. In addition to cell differentiation and pluripotency, self-renewal capacity is another important characteristic that allows CSCs to maintain their growth in a tumor. In contrast with normal proliferation that occurs during the cell cycle, CSCs undergo a distinct cell division process whereby newly generated daughter cells remain in an undifferentiated state and maintain their unique stem cells traits [46]. As a result, CSCs are able to retain their stem cell characteristics throughout cell division.

Self-renewal capacity can be analyzed by two distinct methods. Clonogenic assays evaluate the formation of a colony from a single cell and sphere formation assays examine the growth of spheres in a serum-free sphere medium [29]. In the present study, over-expression of each *TAS2R8* and *TAS2R10* resulted in the suppression of both clonogenic growth and sphere formation. Previously, it was observed that GPCRs exhibit diverse aberrant expression in various cancers and that they regulate stem cells as well as CSCs. In addition, expression levels of GPCRs have been found to strikingly differ in the distinct stages of differentiation of stem cells [47]. In the present study, the role of TAS2R8 and TAS2R10 in the stemness of the NB cancer cells examined suggests their potential as chemotherapeutic targets, despite previously characterizations of TAS2Rs as elusive receptors with distinct signaling pathway and functions compared with other GPCRs.

The self-renewal capacity of CSCs is related to their tumorigenic potential. Here, stable expressions of TAS2R8 and TAS2R10 suppressed tumor incidence and tended to inhibit tumor growth. These observations provided strong evidence that TAS2Rs represent a new type of anti-tumorigenic regulator. Furthermore, a possible mechanism underlying the anti-tumorigenic activities of TAS2Rs appears to involve the neuronal differentiation of BE(2)C cells. Over-expression of TAS2Rs induced the differentiation of NB cells concomitant with up-regulation of β-tubulin III and increased phosphorylation of ERK1/2.

Over-expression of *TAS2R8* and *TAS2R10* significantly inhibited the expression of various CSC markers, including *DLK1*, *CD133*, *Sox2*, and *Notch1*. Previously, *DLK1* was identified as a principal stem cell gene in neuronal cancers based on its ability to suppress phosphorylation of ERK/MEK signaling that is associated with neuronal differentiation [48, 49]. *CD133* is highly expressed in I-type NB cells (such as BE(2)C), and its structural features (including membrane protrusion) influence biological processes such as migration, proliferation, and self-renewal capacity [50–52]. Sox2 suppresses the neural differentiation of progenitor cells, and it acts inversely to proneural transcription factor, *neurogenin-2* [52, 53]. Notch signaling has been shown to modulate tumor development by altering the microenvironment of tumors and cell functions such as adhesion, transition, and proliferation [54]. Therefore, suppression of these CSC markers following over-expression of *TAS2R8* and *TAS2R10* represents another possible anti-CSC mechanism mediated by TAS2Rs.

Decreased oxygen levels, or hypoxia, has been extensively associated with solid tumors, chemotherapy resistance, and enhanced cancer cell stemness [49, 55]. Moreover, hypoxia has been shown to increase cell adhesion, tumor cell invasion and metastasis by enhancing oxygen transportation or by enhancing the adaption of cells to low oxygen conditions in metastatic cancers [56]. HIF-1α is a major regulator of the tumor cell response to hypoxia. Downstream targets of hypoxia-induced, oxygen-dependent HIF-1α include *VEGF* and *GLUT1*, and transactivation of these targets contributes to tumor angiogenesis and glucose metabolism [57]. Hypoxia is also associated with increased MMP activity, which is associated with a poor prognosis for cancer patients [35]. Therefore, targeting of the HIF-1 α is an important strategy for suppressing cancer metastasis.

In the present study, over-expression of *TAS2R8* and *TAS2R10* suppressed the migration and invasion of NB cells, as well as the activation and expression of MMP-2. Moreover, very low levels of MMP-9 activity detected in SK-N-BE(2)C cell using zymography, which was consistent with previous reports (data are not shown) [58, 59]. Neuroblastoma cells express MMP-2 which degrades the basement membrane and they express low or undetectable, levels of MMP-9 [59]. Suppression of HIF-α and its down-stream genes, *VEGF* and *GLUT1*, were also observed following TAS2Rs over-expression. Previously, expression of TAS2Rs was reported to be lower in metastatic breast cancer cells compared to normal epithelial cells [6]. Taken together, these results provide compelling evidence that TAS2Rs have the potential to inhibit angiogenesis, glucose metabolism, and NB cell metastasis via down-regulation of HIF-1α and its downstream targets, *VEGF* and *GLUT1*. However, due to the lack of evidence regarding the effect of TAS2Rs on tumor invasion and metastasis, further studies are needed to confirm these roles both *in vivo* and *in vitro* for different cancers.

Bitter taste has evolved as an important warning signal to protect against the ingestion of potentially toxic food. In a previous study, bitter tasting substances, such as drugs or phytochemicals, were shown to affect drug efflux pumps following the activation of certain receptors, including TAS2Rs. For example, TAS2R38 and other TAS2Rs that were activated by various ligands induced an increase in Ca^2+^ levels, and this promoted the action of efflux transporter ATP-binding cassette B1 (ABCB1) to remove poisonous toxins out of the cells. These results suggest that TAS2Rs may represent drug targets for chemoresistance [8, 60], and further studies of the roles of TAS2R8 and TAS2R10 in relation to drug efflux pumps are warranted.

However, with the recent identification of taste receptors in non-gustatory tissues, the potential for taste receptors to serve as a second line of defense in other tissues should be considered. As such, the identification of harmful drugs and toxins and their role in certain diseases should be investigated. GPCRs have been used as drug targets due to the diverse set of chemicals they can bind, their accessible location on the surface of the cell, the transduction and amplification of their signaling pathways in targeted cells, and their selective expression profiles in different cells. Consequently, multiple therapeutic targeting drugs and therapy strategies have been developed by antagonizing GPCRs [47]. For example, down-regulation of GPR56 has been reported to have various roles in cancer growth both *in vitro* and *in vivo* [61]. However, chemosensory receptor, such as TAS2Rs, are considered to possess distinct properties from the characteristics typical of GPCRs. *TAS2R4* is expressed at higher levels in normal mammary epithelial cells compared with breast cancer cells, and only 5 of the 25 TAS2Rs levels were found to be down-regulated in the invasive breast cancer cell lines that were examined [6].

Among the 25 TAS2Rs that are expressed in humans, *TAS2R8* and *TAS2R10* have not been concrete investigated their physiological roles, unlike the conclusive mechanisms that have been identified for TAS2R38 and TAS2R14. Numerous studies have described taste-related roles for TAS2Rs. However, very few studies have investigated the potential for TAS2Rs to contribute to other physiological aspects and diseases. Bitter compounds and their agonists, including quinine, quinidine, chloroquine, and ally isothiocyanate induced apoptosis and suppressed cell proliferation in breast cancer and bladder cancer [25, 62]. Furthermore, analysis of a glioma genome-wide association dataset identified a possible contribution by a TAS2R8 single nucleotide polymorphism to glioma susceptibility [63], while TAS2R10 has been found to be up-regulated in patients with Parkinson disease and to have a role in the treatment of asthma in rats [64, 65]. These results indicate possible physiologic functions of TAS2Rs, although it has not been confirmed whether the functions of TAS2R8 and TAS2R10 contribute to cancers. Therefore, we selected two subtypes among the currently identified TAS2Rs to investigate their role in neuroblastoma in this present study.

In the present study, both TAS2R8 and TAS2810 exhibited anti-cancer stemness and anti-invasion effects. Recently, a database of bitter compounds (available at http://bitterdb.agri.huji.ac.il/bitterdb/) was established. This database includes over 550 compounds that have been reported to taste bitter to humans [66]. Thousands of bitter tastants and metabolites that possess favorable therapeutic effect and their chemical features may be associated with bitterness. In addition, individual TAS2Rs can have multiple agonists, while one agonist can bind to several TAS2Rs at the same time [4]. Therefore, TAS2Rs may have their own physiological roles in various diseases, and this may involve the differential expression of TAS2Rs according to location and types of receptor. It remains for the mechanisms that mediate TAS2R signaling and functions in relation to cancer stemness, metastasis, and the tumor microenvironment to be confirmed, and for the potential synergism between TAS2Rs to be examined.

In the xenograft model that was established, TAS2R8 mediated a more effective suppression of tumorigenesis than TAS2R10. There are 25 types of bitter taste receptors in humans, and it has recently been discovered that they are expressed in tissues other than the tongue [67]. In the present study, the levels of TAS2R8 and TAS2R10 expression were distinct in different tissues, including liver, tongue, cerebellum, and hypothalamus tissues. It is possible that different receptors may have distinct functions or degrees of effectiveness. Therefore, it will be important for future studies to investigate the function of these receptors in various tissues and to further characterize the discrepancy observed in the present study. Structure-function studies have provided valuable insight into the potential functions of the 25 known TAS2Rs [42, 68]. However, fewer biochemical, pathophysiological, and pharmacological studies have been conducted. It is important that the roles of TAS2Rs in various diseases and in association with other taste receptors be confirmed. Then, studies of novel targeting mechanisms and drug targeting strategies for various diseases in addition to cancer will be facilitated.

In conclusion, to our knowledge, the present study is the first to report evidence that TAS2Rs may suppress cancer stemness by inducing neuronal cell differentiation and by suppressing self-renewal capacity and tumorigenicity in human NB cells *in vitro* and *in vivo*. In addition, the inhibitory effect of TAS2Rs on invasion/migration was demonstrated in relation to suppression of the enzymatic activity and expression of MMPs, including the transcriptional activity of HIF-1α and its downstream genes, *VEGF* and *GLUT1*. Taken together, these novel insights into the molecular mechanisms of TAS2Rs suggest that this class of GPCRs represents a promising cancer therapeutic target to affect the cancer stemness and invasive phenotype of NB cells.
